# Regulation of T Follicular Helper Cells in Islet Autoimmunity

**DOI:** 10.3389/fimmu.2018.01729

**Published:** 2018-07-23

**Authors:** Isabelle Serr, Carolin Daniel

**Affiliations:** ^1^Research Group Immune Tolerance in Diabetes, Institute for Diabetes Research, Helmholtz Diabetes Center at Helmholtz Zentrum München, Munich, Germany; ^2^German Center for Diabetes Research (DZD), Munich, Germany

**Keywords:** T follicular helper cells, islet autoimmunity, microRNA92a, krueppel-like factor 2, type 1 diabetes

## Abstract

T follicular helper (TFH) cells are an integral part of humoral immunity by providing help to B cells to produce high-affinity antibodies. The TFH precursor compartment circulates in the blood and TFH cell dysregulation is implied in various autoimmune diseases including type 1 diabetes (T1D). Symptomatic T1D is preceded by a preclinical phase (indicated by the presence of islet autoantibodies) with a highly variable progression time to the symptomatic disease. This heterogeneity points toward differences in immune activation in children with a fast versus slow progressor phenotype. In the context of T1D, previous studies on TFH cells have mainly focused on the clinically active state of the disease. In this review article, we aim to specifically discuss recent insights on TFH cells in human islet autoimmunity before the onset of symptomatic T1D. Furthermore, we will highlight advances in the field of TFH differentiation and function during human islet autoimmunity. Specifically, we will focus on the regulation of TFH cells by microRNAs (miRNAs), as well as on the potential use of miRNAs as biomarkers to predict disease progression time and as future drug targets to interfere with autoimmune activation.

## Introduction

T follicular helper (TFH) cells are a subset of CD4^+^ T cells characterized by the expression of the C-X-C chemokine receptor type 5 (CXCR5) (1–3) and their master transcription factor B-cell lymphoma 6 (BCL6) (4–6) as well as secretion of the cytokine interleukin-21 (IL-21) (7–9). The expression of CXCR5 together with a low expression of C-C chemokine receptor 7 (CCR7) allows these T cells to enter the B cell follicle in the secondary lymphoid organs (10, 11), where they take part in the germinal center reaction. Specifically, TFH cells interact with germinal center B cells to induce maturation, class switching, and the production of high-affinity antibodies and are therefore an integral part of humoral immunity (1–3).

Although their primary point of action is in the lymph nodes, studies have demonstrated that TFH cell precursors can be found in the blood circulation. These circulating TFH precursors are characterized by the expression of CXCR5, high expression of programmed cell death 1 and low expression of CCR7. Furthermore, circulating TFH precursors are clonally related and phenotypically similar to germinal center TFH cells and comprise a memory compartment that can be reactivated and expanded in response to immunization (12). Therefore, changes in the frequency and phenotype of circulating TFH precursors correlate with those of active TFH cells in the lymph nodes during infections (13). Since continuous stimulation of TFH cells with antigen, in the follicles provided by germinal center B cells, is important to maintain high levels of BCL6 (14), circulating TFH precursors display low or intermediate levels of BCL6 (13).

T follicular helper precursor cells can be subdivided into different subsets according to the effector cytokines they express in parallel to IL-21. Three TFH subsets can be distinguished based on their surface expression of CXCR3 and CCR6. Th1-like TFH cells are CXCR3^+^CCR6^−^ and produce IFNγ, Th2-like TFH cells are CXCR3^−^CCR6^−^ and produce IL-4, IL-5, and IL-13, and Th17-like TFH cells are CXCR3^−^CCR6^+^ and secrete IL-17A and IL-22 (15). Whereas Th2- and Th17-like TFH cells can induce naïve B cells to become plasma cells and produce antibodies, Th1-like TFH cells are suggested to lack this ability (15, 16). CXCR3^+^ TFH precursors were shown to correlate with effective vaccination responses by inducing antibody release from pre-existing memory B cells (16). However, also the memory B cell help by CXCR3^+^ TFH precursors is less efficient compared to that of their CXCR3^−^ counterparts (13, 17). Th2- and Th17-like TFH cells do, however, impact differentially on the class switching of B cells, with Th2-like TFH cells promoting rather IgG and IgE responses and Th17-like TFH cells promoting IgG and IgA responses (15). A recent study on prostate cancer suggests that Th2- and Th17-like TFH cells also impact differentially on the subtype of IgG antibodies produced (18).

Because of their integral role in humoral immunity, TFH cells have been studied in depth in the context of vaccination. Their function of inducing high-affinity antibody responses additionally implies a role of TFH cells in the development and progression of autoimmune diseases that are characterized by the presence of autoantibodies.

One such autoimmune disease is type 1 diabetes (T1D). T1D is the most common metabolic disorder in children and its incidence is rising steadily, especially in young children (19). Impairments in immune tolerance mechanisms can lead to the destruction of the pancreatic insulin-producing β-cells and consequently a failure of blood glucose control, making life-long insulin replacement therapy necessary for patients with symptomatic T1D.

Symptomatic T1D is preceded by a presymptomatic phase (termed islet autoimmunity), characterized by the presence of autoantibodies against islet autoantigens (insulin, insulinoma antigen 2, glutamic acid decarboxylase, zinc transporter 8). The presence of multiple islet autoantibodies increases the life-long risk to develop the symptomatic disease to approximately 100% (20). The time taken for the progression from the development of the first autoantibodies (seroconversion) to the development of the symptomatic disease is, however, very heterogeneous and can range from months (fast progressors) to decades (slow progressors) (20). Accordingly, in our studies, we distinguish different stages of islet autoimmunity: recent onset of islet autoimmunity with islet autoantibodies for less than 5 years and long-term islet autoimmunity with islet autoantibodies for more than 10 years without progression to clinical overt T1D (21–23). However, the immunological mechanisms underlying these differences in disease progression remain poorly understood (24).

## TFH Cells in Presymptomatic T1D

**Figure 1:**
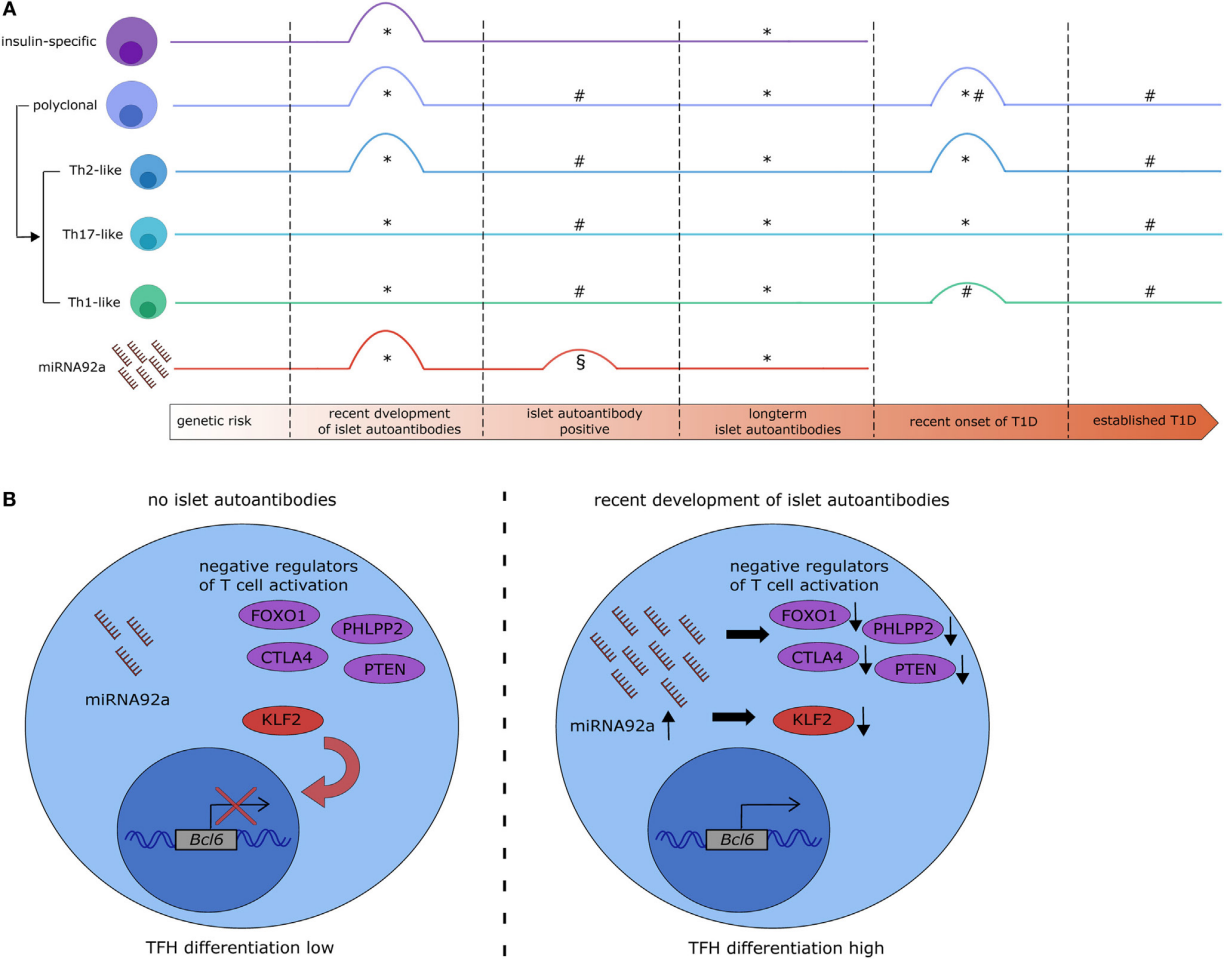
MicroRNA (miRNA)92a expression links alterations in T follicular helper (TFH) precursor frequencies with islet autoimmunity. **(A)** Overview of recent studies on the dynamics of circulating TFH precursor frequencies and miRNA92a abundance in islet autoimmunity. *Serr et al. (22), ^#^Viisanen et al. (32), ^§^Snowhite et al. (33). ^#§^: islet autoantibody positive participants were not stratified based on the duration of islet autoantibody positivity. ^§^: increase in miRNA92a was borderline significant and statistical significance was not reached after additional data processing. **(B)** Potential signaling mechanisms in CD4^+^ T cells targeted by miRNA92a. In states of no islet autoimmunity (left) miRNA92a is expressed at low levels, allowing for the expression of its targets. Targets of miRNA92a are among others negative regulators of T cell activation (e.g., FOXO1, PHLPP2, CTLA4, and PTEN) and negative regulators of TFH differentiation [e.g., krueppel-like factor 2 (KLF2)] which contributes to a reduced expression of the TFH transcription factor B-cell lymphoma 6 (BCL6) and reduced TFH differentiation. During recent onset of islet autoimmunity (right) the expression of miRNA92a is upregulated, leading to a decreased expression of its targets, increased expression of BCL6, and increased TFH differentiation.

Alterations in the frequency or function of TFH precursor populations in the peripheral blood have been implicated in various autoimmune disorders, including systemic lupus erythematosus and T1D (25–27). Regarding T1D, Kenefeck et al. have demonstrated in a transgenic TCR model that the transfer of TFH cells can induce diabetes. Specifically, they transferred ovalbumin-specific CXCR5^+^ or CXCR5^−^CD4^+^ T cells into recipient mice expressing ovalbumin under the insulin promoter in the β-cells and observed a significant increase in diabetes incidence in mice receiving CXCR5^+^CD4^+^ T cells (28). Furthermore, Ferreira et al. observed increased IL-21 production by CD4^+^ T cells in T1D patients (29). These previous studies on TFH cells in T1D have focused on symptomatic T1D, which excludes conclusions regarding the involvement of TFH cells in the presymptomatic phase or the progression to clinical T1D. The development of multiple islet autoantibodies characterizes the onset of presymptomatic T1D. The important contribution of TFH cells to humoral immunity therefore implicates an involvement of these cells also in disease onset and progression. Accordingly, we found insulin-specific and polyclonal TFH precursor frequencies to be increased during recent onset of islet autoimmunity. This increase was, however, transient and in children with long-term islet autoimmunity without progression to symptomatic T1D, the TFH precursor frequency was similar to that observed in children without islet autoantibodies (22) (Figure 1A). This is in accordance with the observation that children with long-term islet autoimmunity tend to lose their first islet autoantibodies, most commonly insulin autoantibodies (30). Data from birth cohort studies highlight that proinsulin-specific CD4^+^ T cells of children who developed islet autoantibodies show a gene expression signature resembling TFH/TH17 cell responses already very early on in infancy, well before the development of islet autoantibodies (31). In a recent Finnish study, no alterations in circulating TFH precursors were observed in normoglycemic children with multiple islet autoantibodies (32) (Figure 1A). However, study participants were not discriminated according to the duration of islet autoantibody positivity. These seemingly divergent results highlight the heterogeneity of T1D and underline the necessity to more precisely discriminate the stages of islet autoimmunity and age of study participants.

Regarding the function of circulating TFH precursors, the analysis of Th1-, Th2-, and Th17-like TFH precursors is relevant, because of differences in their ability to provide B cell help and impact on antibody isotype production (15). Data regarding TFH precursor subsets in autoimmune diseases is limited; however, we reported an increase specifically in the Th2-like TFH subset in children with recent onset of islet autoimmunity and in children with newly diagnosed clinical T1D, whereas Th1- and Th17-like TFH cells were unaltered (22). Although Ig subtypes were not analyzed in our study, previous studies highlighted that Ig isotypes of islet autoantibodies and even IgG subtypes induced in the presymptomatic phase of the disease might influence the disease progression (34–36). Similarly, regarding autoimmune diseases other than T1D, a study by Le Coz et al. highlighted an increase of Th2-like TFH cells, accompanied by a decrease in Th1-like TFH cells in patients with systemic lupus erythematosus (37). In this study, Le Coz et al. demonstrate that IgE levels in the serum of lupus patients correlate with disease activity and are associated with high frequencies of Th2-like TFH cells (37).

## Mechanisms of TFH Induction in Islet Autoimmunity

The TFH differentiation process is highly complex, involving several steps and factors (25–27). In 2013, two research groups demonstrated an important role of the microRNA17~92 (miRNA17~92) cluster, which is essential for normal TFH development and function in mice (38, 39). miRNAs are small, ~22 nucleotide long, non-coding RNAs which can complementarily bind their target mRNAs in the RNA-induced silencing complex and induce their translational silencing or degradation (40–42). miRNAs usually have a multitude of targets and induce rather modest regulation (43, 44), enabling them to regulate complex cellular states, such as T cell activation (45, 46) and making them suitable targets for immune modulating therapies.

The miRNA17~92 cluster transcribes six mature miRNAs (miRNA17, miRNA18a, miRNA19a, miRNA19b, miRNA20a, and miRNA92a). The relevance of these miRNAs in autoimmune diseases is highlighted by the fact that overexpression of the cluster leads to autoimmunity and autoantibody production in mice (47). Regarding the role of the cluster in murine TFH cell differentiation, miRNA17~92 regulates differentiation and migration of TFH cells together with Bcl6 by repressing TFH subset inappropriate genes like *retinoid-related orphan receptor α* (*Rora*) and by regulating signaling molecules important for TFH differentiation and function, such as inducible T cell costimulator (Icos) and phosphatidylinositol-3-kinase (PI3K)/protein kinase B signaling (38, 39). Accordingly, two validated targets of miRNA92a are the *phosphatase and tensin homolog* (*Pten*) and *PH domain and leucine rich repeat protein phosphatase 2* (*Phlpp2*), both negative regulators of PI3K signaling (47, 48). In line with its role in TFH cell differentiation and function, additional confirmed targets for miRNA92a are other negative regulators of T cell activation, such as *forkhead box protein O1* (*Foxo1*) and *cytotoxic T-lymphocyte associated protein 4* (*Ctla4*) (47, 48).

In an miRNA profiling approach to investigate miRNAs in T cells that could be involved in human autoimmune activation, we identified miRNA92a to be significantly increased in CD4^+^ T cells from children with ongoing islet autoimmunity compared to healthy controls (22). Confirmation *via* RT-qPCR highlighted an increase in miRNA92a specifically in T cells from children with recent onset of islet autoimmunity and not in children with long-term islet autoimmunity (Figure 1A). Our analysis demonstrated furthermore that this increase in miRNA92a expression correlates with TFH precursor frequencies in the peripheral blood. Accordingly, the lowest expression of miRNA92a was found in T cells from children with long-term islet autoimmunity.

**Figure 2:**
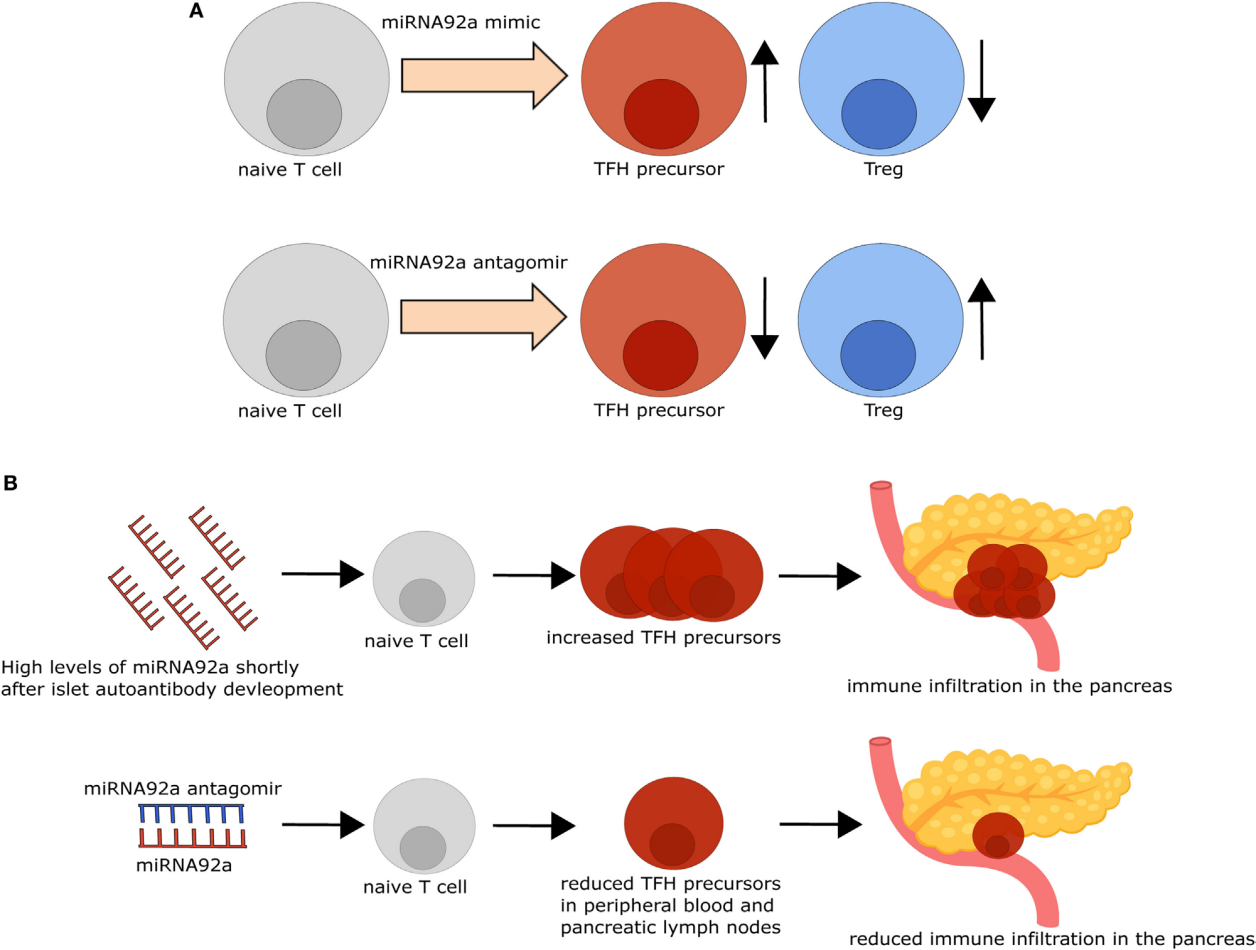
Modifying microRNA (miRNA)92a activity impacts T follicular helper (TFH) and regulatory T cell (Treg) induction *in vitro* and immune activation *in vivo*. **(A)** TFH induction *in vitro* is increased, while Treg induction *in vitro* is decreased in the presence of an miRNA92a mimic (upper row). By contrast, inhibition of miRNA92a, using an miRNA92a antagomir (lower row), results in decreased TFH induction and increased Treg induction *in vitro*. **(B)** Non-obese diabetic (NOD) mice with islet autoantibodies express increased amounts of miRNA92a and increased TFH precursor frequencies, accompanied by immune infiltration in the pancreas. Treatment of NOD mice with an miRNA92a antagomir reduces circulating TFH precursor frequencies and immune cell infiltration in the pancreas.

For the investigation of the role of miRNA92a in human TFH differentiation, *in vitro* TFH induction assays, relying on the stimulation of human naïve CD4^+^ T cells with anti-CD3 and anti-CD28 antibodies in the presence of memory B cells, were established. In line with a role of miRNA92a in TFH induction, human TFH induction was decreased in *in vitro* assays, when miRNA92a activity was blocked, whereas an miRNA92a mimic promoted TFH induction (Figure 1B). In assays with an miRNA92a mimic, negative regulators of T cell activation such as *PTEN, PHLPP2, FOXO1*, and *CTLA4* that are confirmed targets of miRNA92a, were reduced in their expression (22) (Figure 1B). These findings are in line with previous studies, highlighting that TFH cell differentiation is largely dependent on low levels of FOXO1, maintained either by ICOS-PI3K signaling or by degradation *via* the E3 ubiquitin ligase ITCH (49, 50). miRNA92a mediated TFH induction likewise depends on PI3K signaling, since *in vitro* TFH induction with an miRNA92a mimic is blunted in the presence of a PI3K inhibitor, whereas it is increased when PTEN is inhibited (22). PTEN, as a negative regulator of PI3K signaling, is critically involved in the *de novo* induction of regulatory T cells (Tregs). Accordingly, *in vitro* Treg induction from naïve CD4^+^ T cells was found to be impaired in the presence of an miRNA92a mimic. Moreover, insulin-specific Treg frequencies are reduced in children with recent onset of islet autoimmunity, a disease state where miRNA92a abundance was shown to be significantly enhanced in T cells (21, 22) (Figure 2A).

T follicular helper cell function is largely dependent on their ability to enter the B cell follicle in the lymph nodes. Therefore, molecules that regulate lymphocyte trafficking and homing are important mediators of TFH cell function. One example is krueppel-like factor 2 (KLF2). Lee et al. demonstrated that TFH differentiation is dependent on low levels of Klf2, since Klf2 induces the expression of *sphingosine 1 phosphate receptor 1* (*S1pr1*), which opposes TFH induction (51). Furthermore, Klf2 was shown to inhibit *Bcl6* expression by upregulating B-lymphocyte induced maturation protein 1 (51). Interestingly, our data suggest that *KLF2* can be directly targeted by miRNA92a, since a target site blocker, that inhibits the binding of miRNA92a specifically to *KLF2* abolishes *in vitro* TFH induction (22) (Figure 1B), thereby offering one additional mechanism of miRNA92a-mediated TFH differentiation.

## miRNAs as Biomarkers in Islet Autoimmunity

The heterogeneous disease progression from the development of islet autoantibodies to the symptomatic disease necessitates the discovery of biomarkers that will enable a better prediction of the progression time to the clinically active disease. To that end, it remains to be determined, whether changes in miRNA92a expression can also be observed in the serum of children with recent development of islet autoantibodies, or whether the detection of these alterations is limited to the CD4^+^ T cell population. One recent study by Snowhite et al. aimed at identifying differentially expressed miRNAs in the serum of children with and without autoantibodies. miRNA92a was one of the identified miRNAs that was increased in children with autoantibodies, however, this increase was only borderline significant and not significant after further data processing (33) (Figure 1A). The autoantibody positive children investigated in this study were not stratified based on the duration of autoantibody positivity, which might account for this outcome. The study of longitudinal samples from children at risk of developing T1D will help to assess the usefulness of miRNA92a, TFH cell frequencies, and their respective subsets as biomarkers to predict the progression to clinically overt T1D. More specifically, the analysis of possible correlations between these markers and autoantibody titers or subtypes might be of interest. In this context, a correlation of miRNA92a expression in T cells with TFH precursor frequencies in the blood as well as a modest correlation with insulin autoantibody titers was reported (22). A more detailed analysis of TFH precursor subsets might be especially relevant, because of their divergent functions with respect to providing B cell help and impacting Ig subtype production. Moreover, given the negative impact of high miRNA92a levels on Treg induction, the analysis of Treg frequencies and possible inverse correlations with miRNA92a abundance or TFH subset frequencies can be envisioned. Together, these analyses could be useful to define TFH signature profiles that might serve as biomarkers for assessing T1D disease progression.

## Targeting miRNAs to Interfere with Autoimmune Activation

miRNAs can function as promising novel potential drug targets, since they can be targeted by small, highly specific oligonucleotides. In this regard, clinical trials for the treatment of hepatitis C virus infections with an miRNA inhibitor have been successfully conducted (52). Targeting specific cell types, especially immune cells, with miRNA inhibitors is, however, challenging, because of the negative charge of the oligonucleotides which inhibits penetration of the cell membrane (53). Research efforts focus mainly on encapsulation techniques, and various nanoparticles were shown to mediate an efficient uptake of small RNAs by lymphocyte populations (54). Other techniques, targeting T cells more specifically, are, e.g., the use of a single chain CD7 antibody (scFvCD7) fused to an oligonucleotide-nona-arginine peptide (55).

The possibility of altering immune activation and regulation by targeting miRNAs was demonstrated in insulin autoantibody positive non-obese diabetic mice, the most commonly used mouse model for T1D. *I.p*. Application of an miRNA92a antagomir, optimized for *in vivo* use, decreased TFH frequencies and immune activation in the pancreas, accompanied by decreased insulitis scores and autoantibody titers (22). Furthermore, this decreased immune activation went along with increased frequencies of Tregs in treated animals, suggesting that, apart from reducing immune activation, inhibition of miRNA92a positively impacts on mediators of T cell tolerance (Figure 2B).

The restoration of immune tolerance mechanisms in autoimmune diseases is a long envisioned goal. Since Tregs are important mediators of T cell tolerance in the periphery and can be induced in an antigen-specific fashion, Treg induction could contribute to interfering with the progression of autoimmune activation in autoimmune diseases. This notion is supported by identified associations indicating high frequencies of insulin-specific Tregs accompanied by reduced numbers of insulin-specific TFH precursors in the peripheral blood of children with long-term islet autoimmunity without progression to clinically active T1D. During recent onset of islet autoimmunity, a significant decrease in insulin-specific Treg frequencies was observed accompanied by impaired *in vitro* Treg induction (23). Specifically, during this critical time frame we found an increased sensitivity to antigenic stimulation in naïve CD4^+^ T cells and reduced expression of negative regulators of T cell activation which can interfere with efficient Treg induction (23). Using miRNAs to tame T cell activation during ongoing islet autoimmunity might therefore open a window of opportunity for improving Treg induction potential in a setting, where the autoimmune process is already in progress. However, the effectiveness of inhibiting miRNA92a to interfere with autoimmune activation and progression to T1D requires long-term *in vivo* studies in animal models of T1D, which are missing so far.

## Conclusion

Accumulating evidence points toward a role of TFH cells in the development of autoimmune diseases including T1D. During recent onset of islet autoimmunity, children display increased frequencies of TFH precursor cells, specifically Th2-like TFH precursors, whereas this increase is absent in children with long-term islet autoimmunity without overt T1D (22). The analysis of TFH cell frequencies or miRNAs involved in TFH development in longitudinal samples could therefore help to identify biomarkers in order to improve our ability to predict the progression time to clinically overt T1D, as well as to improve the stratification of respective disease groups. In addition, progress is made regarding the cell type-specific delivery of miRNA inhibitors or mimics. Since miRNAs regulate cellular states, rather than single targets, they compose a new, promising group of future drug targets. In this regard, miRNAs such as miRNA92a that regulate TFH differentiation and function might be targeted to limit immune activation in settings of autoimmunity such as T1D.

## Author Contributions

IS wrote the manuscript and designed illustrations. CD conceptualized, wrote, and edited the manuscript.

## Conflict of Interest Statement

The authors declare that the research was conducted in the absence of any commercial or financial relationships that could be construed as a potential conflict of interest.
